# Identification and Characterization of *TALE* Homeobox Genes in the Endangered Fern *Vandenboschia speciosa*

**DOI:** 10.3390/genes8100275

**Published:** 2017-10-17

**Authors:** Mercedes Ruiz-Estévez, Mohammed Bakkali, Rubén Martín-Blázquez, Manuel A. Garrido-Ramos

**Affiliations:** Departamento de Genética, Facultad de Ciencias, Universidad de Granada, 18071 Granada, Spain; mercy_canaria@hotmail.com (M.R.-E.); mbakkali@ugr.es (M.B.); rmblazquez@ugr.es (R.M.-B.)

**Keywords:** *TALE* homeobox genes, *KNOX* genes, *BELL* genes, fern, *Vandenboschia speciosa*, expression patterns, qRT-PCR, transcriptome

## Abstract

We report and discuss the results of a quantitative reverse transcription polymerase chain reaction (qRT-PCR) analysis of the expression patterns of seven three amino acid loop extension (*TALE*) homeobox genes (four *KNOTTED-like homeobox* (*KNOX*) and three *BEL1-like homeobox* (*BELL*) genes) identified after next generation sequencing (NGS) and assembly of the sporophyte and gametophyte transcriptomes of the endangered fern species *Vandenboschia speciosa*. Among the four *KNOX* genes, two belonged to the *KNOX1* class and the other two belonged to the *KNOX2* class. Analysis of the deduced amino acid sequences supported the typical domain structure of both types of TALE proteins, and the homology to TALE proteins of mosses, lycophytes, and seed plant species. The expression analyses demonstrate that these homeodomain proteins appear to have a key role in the establishment and development of the gametophyte and sporophyte phases of *V. speciosa* lifecycle, as well as in the control of the transition between both phases. *Vandenboschia speciosa* VsKNAT3 (a KNOX2 class protein) as well as VsBELL4 and VsBELL10 proteins have higher expression levels during the sporophyte program. On the contrary, one *V. speciosa KNOX1* protein (VsKNAT6) and one KNOX2 protein (VsKNAT4) seem important during the development of the gametophyte phase. *TALE* homeobox genes might be among the key regulators in the gametophyte-to-sporophyte developmental transition in regular populations that show alternation of generations, since some of the genes analyzed here (*VsKNAT3*, *VsKNAT6*, *VsBELL4*, and *VsBELL6*) are upregulated in a non-alternating population in which only independent gametophytes are found (they grow by vegetative reproduction outside of the range of sporophyte distribution). Thus, these four genes might trigger the vegetative propagation of the gametophyte and the repression of the sexual development in populations composed of independent gametophytes. This study represents a comprehensive identification and characterization of *TALE* homeobox genes in *V. speciosa*, and gives novel insights about the role of these genes in fern development.

## 1. Introduction

The phylogenetic position of ferns as the sister lineage of seed plants makes the study of developmental genes in these vascular plants highly useful, and necessary to open new insights on plant evolutionary developmental biology [1,2,3,4,5,6,7]. *Vandenboschia speciosa* is a rare vulnerable European-Macaronesian endemism, the only representative of a genus which has a primarily tropical distribution, restricted to disjoined regions of the European Atlantic coast and the Macaronesian islands (Canaries, Madeira and Azores). These regions are tertiary flora refuges harbouring relic populations that survived the glacial cycles. The two phases of *V. speciosa* life cycle are perennial, and can reproduce by vegetative propagation [8]. The “floaty” sporophyte (fronds made of a translucent single layer of cells) is rhizomatous, and can spread by fragmentation of the rhizome. The gametophyte, unlike a heart-shaped prothallus, is epigeous and narrowly filamentous with specialized asexual propagules (gemmae). While the sporophyte is adapted to growth in areas with low incidence of light and constant moisture, the gametophyte can live in a wider range of habitats, including less humid and darker ones, and can survive in very sheltered stable microsites, such as caves and deep crevices [8]. Such habitats can provide a microclimate and a stable environment for survival during long periods of independent gametophytes, outside the range of sporophyte distribution [8]. The prolonged gametophyte survival could be facilitated by a low metabolic rate and an ability to make efficient use of the limited available light, thanks to morphological and physiological adaptations [9,10]. In the warmer climatic conditions in the South of the Iberian Peninsula, the Azores, the Madeira, and the Canaries, this species usually undergoes a normal fern lifecycle of two free-living generations, but, as one goes further north and east of Europe, the sporophyte generation becomes increasingly rare [8]. However, one out of seven populations of this species located in the south of the Iberian Peninsula shows only the gametophyte phase. All these features make this species an attractive species for the analysis of different developmental and evolutionary topics, including the ones with important implications for conservation biology. In this context, it is highly interesting to unravel and understand the genetics behind the control of the alternation of the two fern lifecycle phases by characterizing and analyzing homeobox genes, an issue for which the analysis of gametophyte independent populations of *V. speciosa* might represent an important advance.

Homeobox genes encode a typical DNA-binding domain known as homeodomain (HD) that characterizes a large family of transcription factors. In plants, fourteen distinct classes of homeobox genes are found [11,12,13]. The three amino acid loop extension (*TALE*) homeobox gene superclass is characterized by encoding three extra residues (proline–tyrosine–proline) within the loop between the helix 1 and helix 2 of the homeobox-encoded homeodomain [11,12,13,14,15,16,17]. The *TALE* homeobox genes are very ancient, being present in a wide range of eukaryotic kingdoms with five classes in animals and two classes in plants, the *KNOTTED-like homeobox* (*KNOX*) and *BEL1-like homeobox* (*BELL*) genes. Both plant *TALE* genes encode large bipartite domains upstream of the homeodomain, termed *KNOX* and *BEL* domains, respectively. In addition, the *KNOX* genes encode the *ELK* domain between the KNOX and the HD domains. The *BELL* genes additionally encode also the SKY domain and the ZIBEL domains. BELL and KNOX proteins are structurally and functionally related, and have been shown to interact through their BELL and KNOX domains, respectively [14,16,18,19,20,21,22,23]. BELL and KNOX transcription factors appear to be key for the distinctive gametophytic and sporophytic developmental programs, as well as for the gametophyte-to-sporophyte developmental transition that characterizes plants [21,22,23,24,25]. The diversification of KNOX/BELL modules during land plant evolution facilitated the evolution of ever more complex diploid sporophyte body plans [21,23,25].

Divergence between *KNOX* and *BELL* gene lineages occurred before the split between red and green algae [22,25]. Green algae have a single *KNOX* and a single *BELL* gene. Prior to the origin of land plants, a gene duplication in an ancestral *KNOX* gene generated two classes of *KNOX* genes, class I (*KNOX1*) and class II (*KNOX2*) [11,22,25,26,27], increasing fundamental structural and developmental challenges related to the transition to land [22,28,29,30,31]. In fact, the *KNOX* genes have experienced two major events of expansion during the evolution of plants, one from algae to moss and the other during the transition from lycophytes to angiosperms [22]. There are five *KNOX* genes in bryophytes (three *KNOX1* and two *KNOX2*) and four *KNOX* genes in lycophytes (two *KNOX1* and two *KNOX2*), while there has been a diversification of *KNOX* genes in vascular plants [11,14,18,22,25,32]. In *Arabidopsis thaliana*, there are eight *KNOX* genes (four *KNOX1* and four *KNOX2* genes) [22]: *SHOOT MERISTEMLESS* (*STM*), *KNAT1*, *KNAT2*, and *KNAT6* are *KNOX1* genes, while *KNAT3*, *KNAT4*, *KNAT5*, and *KNAT7* are *KNOX2* genes. There are nineteen *KNOX* genes in poplar and fourteen genes in rice [11,23,25]. The diversification of *BELL* genes has also accompanied the evolution of land plants. There are four *BELL* genes in mosses and thirteen *BELL* genes in *A. thaliana* and in potato, while there are fifteen in poplar and twelve in rice [11,17,23,25,33].

In general, there is unfortunately little information about developmental genes in ferns. Furthermore, the genomic data from fern species are not extensive [34], and although new insights are appearing from recent efforts on the genomics of ferns [35,36,37], only a few studies focused on the analysis of individual *KNOX* genes from fern species [27,38,39,40]. In this paper, we report and discuss the results of a quantitative PCR analysis of the expression patterns of four *KNOX* and three *BELL* genes identified after next generation sequencing (NGS) and assembly of the transcriptomes of the sporophyte and of the gametophyte of the endangered fern species, *V. speciosa*. The results are compared between genes, phases, and populations, in order to investigate the different expression patterns between populations with and without alternation of generations, and infer on the role of those genes in fern development. 

## 2. Materials and Methods

### 2.1. Sample Collection

We collected sporophyte, spore filled sporangia, and gametophyte of *V. speciosa* from the population of Valdeinfierno (VALD), located at the Natural Park of Alcornocales (Cádiz, Spain). For comparisons, sporophyte and gametophyte of *V. speciosa* from a second population (Canuto de Ojén Quesada (OJEN)) were also collected. VALD and OJEN are two populations with a regular fern lifecycle of two free-living generations, gametophyte and sporophyte. In addition, gametophytes of this species from La Almoraima (ALMO) were also collected. The ALMO population is composed only of independent gametophytes. The sporophytes were frozen in liquid nitrogen immediately at the moment they were collected. The patches with gametophytes were taken in a Petri dish with soil to the laboratory, where, under a binocular microscope, the filaments were separated from the soil and from other plant species, cleaned, and frozen in liquid nitrogen. All samples were stored at −80 °C for RNA extractions.

### 2.2. Next Generation Sequencing, Transcriptome Assembly, Annotation, and Comparative In Silico Analysis of Gene Expression Levels

For transcriptome sequencing, RNA was isolated from five gametophytes and five sporophytes from VALD (April) using Spectrum Plant Total RNA Kit (Sigma, Madrid, Spain). Pools of sporophyte and gametophyte RNA were generated from the two sets of five individual RNAs and separated. Next generation sequencing of the two samples was carried out (Macrogen Inc., Seoul, Korea) based on the Illumina HiSeq 2000 Paired-end approach (Illumina Inc., Seoul, South Korea).

After assessment of the quality of the resulting sequencing reads, using FastQC [41], ABySS [42] was used to carry out a first multi k-mer transcriptome assembly step using all the sequencing reads in FASTQ format. The k-mers included all the odd numbers from 19 to 95.

A first set of one transcriptome per k-mer was thus generated, and a second assembly step was then carried out in order to merge all these transcriptomes into one, filter the sequences into the merged file to remove redundancy, and extend the remaining non-redundant sequences. This second step was based on the Trans-ABySS pipeline [43], and allowed us to further assemble the reference transcriptome and get the transcripts as lengthy as possible. A final assembly step was also added in order to further extend the assembled transcripts, this time using the CAP3 software [44].

The sequences of the resulting FASTA file were then BLASTed against a locally built database with all the *Arabidopsis*, fern, and moss proteins from the NCBI database. For that, local BLASTX searches were carried out [45], and the hits were considered positive at a 10^−6^ *e*-value threshold. Blast2GO software [46] was then used for functional annotation of the Basic Local Alignment Search Tool (BLAST) positive sequences. 

For comparison of the expression levels, both BLAST positive and BLAST negative sequences were taken as reference transcriptome in annotated FASTA format. The raw sequencing reads (the FASTQ files) from the libraries of the sporophytes and the gametophytes were then separately aligned to the reference sequences using BWA [47]. The number of reads that aligned to each reference sequence was counted using the *htseq-count* script of the HTSeq program [48]. For fold change-based comparison of the number of reads aligned to each sequence between the two libraries, and we carried out normalization of the read counts into Reads per Kilobase of the reference sequence per Million reads in the library (RPKM). Assessment of the statistical significance of the differences was based on a contingency χ^2^-test strategy and false discovery rate correction [49].

### 2.3. Sequence Analysis

BioEdit software (version 7.1.3) [50] was used to deduce the amino acid sequences from the NGS assembled contigs that were earlier annotated as sequences corresponding to homeobox genes. BLAST [45] was then used to search in the protein databases with the deduced amino acid sequences as queries. Sequences from other plants were collected from gene databases. Multiple alignments of deduced amino acid sequences were performed using ClustalX (default parameters), which implement the ClustalW algorithm [51], and adjusted manually afterwards.

### 2.4. Expression Analysis

#### 2.4.1. RNA Extraction and Complementary DNA Synthesis

Total RNA was extracted from sporophytes and gametophytes using the Spectrum Plant Total RNA Kit (Sigma). After RNA extraction, the samples were submitted to a DNase I treatment (REAL Star Kit, Durviz, Paterna, Spain), to ensure the absence of contaminating genomic DNA traces. RNA quantity and purity were measured using Tecan’sInfinite 200 NanoQuant. For each sample, 100 ng RNA was retrotranscribed into complementary DNA (cDNA) using the combination of random and oligo deoxythymidine (dT) primers of the PrimeScript RT reagent—Perfect Real Time-Kit (Takara) and following manufacturer’s recommendations. For each analysis, a negative control devoid of reverse transcriptase was included to test for contaminating DNA.

#### 2.4.2. Target and Housekeeping Genes

The homeobox and housekeeping (*HKG*) genes of *V. speciosa* were identified using BLASTN searches of other plant homeobox and *HKG* sequences against our local *V. speciosa* transcriptome library. The *HKG* sequences searched were those provided by Le Bail et al. [52]. Primers were designed using Primer3 software [53] (Table S1; Supplementary material). We tested their specificity and reliability by PCR amplification and posterior sequencing. PCR reactions mixture contained 30 ng complementary DNA (cDNA), 200 mM deoxynucleotide triphosphates (dNTPs), 10 mM each forward and reverse primers, 2 mM MgCl_2_, and 1 unit of Horse-Power-Taq DNA polymerase (Canvax, Granada, Spain) in a final volume of 20 μL. PCR assays were run in a GeneAmp PCR system 2700 thermocycler (Applied Biosystems, Madrid, Spain), and PCR conditions were as follows: an initial denaturation at 94 °C for 5 min; and 35 cycles of 94 °C for 30 s, 55 °C for 20 s, 72 °C for 20 s; and final extension of 72 °C for 4 min. PCR products were visualized after electrophoresis in 1.5% agarose gel, and cleaned with illustra GFXPCR DNA and Gel Band Purification Kit (GE Healthcare, Madrid, Spain). The PCR products were sequenced at the Centro de Instrumentación Científica (University of Granada), and analyzed using BioEdit software (version 7.1.3) [50].

#### 2.4.3. Relative Gene Expression Quantification

After ensuring the specificity of the target and *HKG* primers, we determined the most stable *HKG*s in our samples through carrying out a geNorm analysis [54]. Standard curve analysis was used to determine the efficiency of the selected *HKG*s. We then estimated the relative expression level of the homeobox genes in the different samples by means of quantitative reverse transcription polymerase chain reaction (qRT-PCR). The reaction mixtures contained 5 μL 2X SensiMix SYBR Mastermix (SensiMix SYBR Kit, Bioline, Barcelona, Spain), 0.35 μM each forward and reverse primer, and 5 ng cDNA, in a final volume of 15 μL. We amplified the same calibrator sample (comprising cDNA synthesized from RNA of different samples) in each run to ensure that the data resulting from the experimental samples were comparable. qRT-PCR assays were run in a Chromo4 Real Time PCR thermocycler (BioRad, Alcobendas, Madrid, Spain), and PCR conditions were as follows: an initial denaturation at 95 °C for 10 min; 40 cycles of 94 °C for 30 s, 55 °C for 20 s, 72 °C for 20 s; and a melting curve step to check the specificity of the reaction. We included a negative control without cDNA to ensure that the reagents were free of contaminating DNA. There was no amplification from this sample, and therefore, the test allowed us to determine that the primers (or any PCR reagent) were not contaminated, nor had the primers formed dimers with each other. Where possible, reagents were combined in master mix solutions to minimize the number of manipulations, and each sample was amplified in triplicate. Opticon Monitor v3.1. software (BioRad) was used to export the qRT-PCR raw data from the Chromo4 instrument and relative quantification (RQ) values of the transcripts were obtained following the “Efficiency calibrated mathematical method for the relative expression ratio in real-time PCR” (Roche Applied Science, Technical Note No. LC 13/2001). This method allows you to use more than one *HKG*s to normalize the C_t_ values and calculate the RQs, employing the geometric average of the Q the *HKG*s used. We identified the specificity of the amplified products by sequencing the qRT-PCR products.

#### 2.4.4. Statistical Analyses

All homeobox gene RQs failed to fit a normal distribution (tested by the Shapiro–Wilk’s test), and thus, the nonparametric Mann–Whitney *U* test was used to analyze them. All these analyses were performed using the STATISTICA 8.0 software (TIBCO Software Inc., Palo Alto, CA, USA) [55].

## 3. Results

### 3.1. Identification and Sequence Analysis of TALE Homeobox Genes Contigs in V. Speciosa

Examination of the two NGS assembled transcriptomes of *V. speciosa* revealed 11 contigs corresponding to 11 putative *KNOX* genes: 3 belonged to the *KNOX1* class and 8 belonged to the *KNOX2* class. In addition, 11 putative BEL1-like type genes were detected. We selected four *KNOX* genes (two of each class) and three *BELL* genes for further analysis. In the case of *KNOX* genes, the selected genes were identified, according their origin and homology, as Vs*KNOX1* and Vs*KNOX2* class gene and named *VsKNAT1*, *VsKNAT3*, *VsKNAT4*, and *VsKNAT6*, according to their homology to different types of *KNOX* (*KNAT*) genes in *A. thaliana*. In the case of *BELL* genes, they were named as *VsBELL4*, *VsBELL6*, and *VsBELL10*, according to their homologous partners in *A. thaliana*. The sequences (Figure S1; Supplementary material) were deposited in the publicly accessible repository of the European Nucleotide Archive (ENA) of the European Bioinformatics Institute (EMBL-EBI).

**Figure 1:**
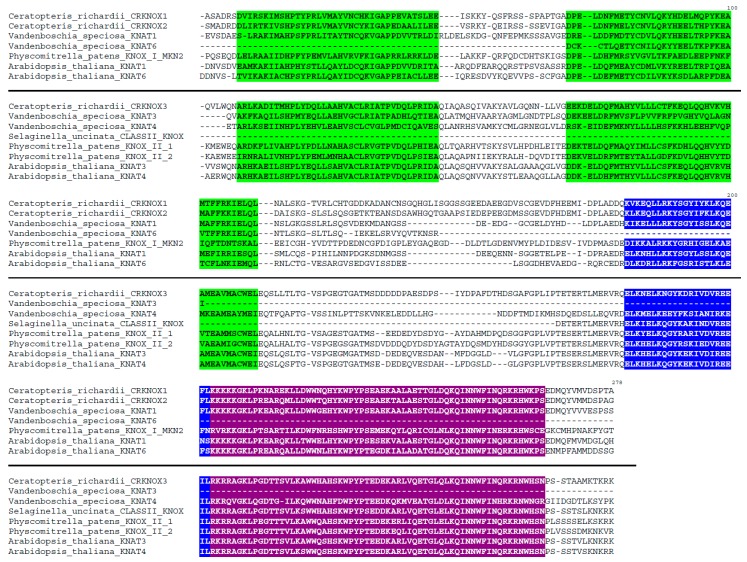
Alignment of the deduced amino acid sequences of the *Vandenboschia speciosa KNOTTED-like homeobox* 1 and 2 (VsKNOX1 and VsKNOX2) proteins with those of other related KNOX proteins of different plant species: the fern *Ceratopteris richardii*, the moss *Physcomitrella patens*, and the seed plant *Arabidopsis thaliana*. The sequences above the line belong to the class KNOX1 of KNOX proteins, whereas the sequences the sequences below the line belong to the class KNOX2. Green boxes mark the KNOX domains, the blue box marks the ELK domain, and the purple box marks the homeodomain.

**Figure 2:**
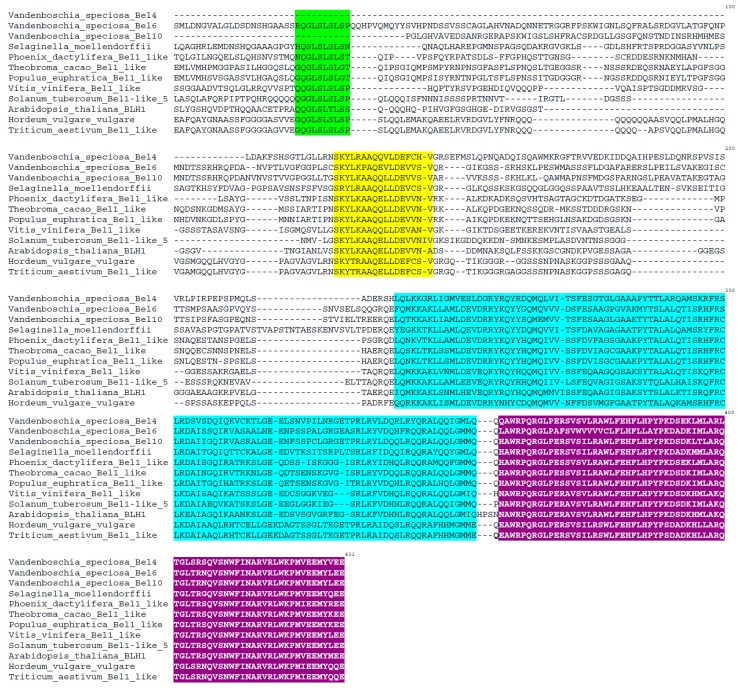
Alignment of the deduced amino acid sequences of the VsBELL proteins from *Vandenboschia speciosa* with those of other related BELL proteins of different plant species: the lycophyte *Selaginella moellendorffii* and several seed plants. Boxes mark the different protein domains: the ZYBEL domain (green), the SKY domain (yellow), the BEL domain (blue), and the three amino acid loop extension homeodomain (purple).

The identity of each VsKNAT and VsBELL amino acid deduced sequences to its homologous proteins from different plant groups, as revealed by BLAST search, is indicated in Table 1. Homologous KNOX sequences found in GeneBank/EMBL databases [56,57] belonged to the moss *Physcomitrella patens*, the fern species *Ceratopteris richardii* and *Elaphoglossum peltatum*, and to several spermatophyte species (Figure 1 and Table 1). Homologous BELL sequences found in GeneBank/EMBL databases [56,57] belonged to the lycophyte species *Selaginella moellendorffii* and *Selaginella kraussiana*, and to several spermatophyte species (Figure 2 and Table 1).

Sequence alignments show a typical KNOX structure for the deduced VsKNAT1 and VSKNAT4 amino acid sequences of *V. speciosa*, which consist of the large bipartite KNOX domain, the ELK domain, and the homeodomain, the latter characterized by having three extra residues, PYP, in the loop between helix 1 and helix 2 of the homeodomain (Figure 1). However, the sequences obtained for VsKNAT3 and VsKNAT6 only covered part of the bipartate KNOX domain, and did not include the other domains. The sequence alignments (Figure 1) clearly defined the VsKNAT1 and VsKNAT6 as *KNOX1* class genes, while VsKNAT3 and *VsKNAT4* belonged to the *KNOX2* class, as previously identified by the BLAST search. Sequence alignments also indicated that the deduced VsBELL amino acid sequences have a typical structure consisting of the ZYBEL domain, the SKY domain, the BEL domain, and the three amino acid loop extension homeodomain (Figure 2).

### 3.2. Expression Analysis of TALE Homeobox Genes in V. speciosa

**Figure 3:**
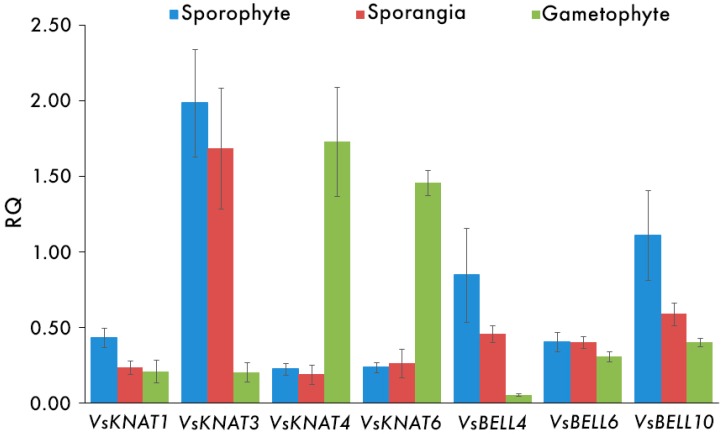
Graphic representation of the mean RQ values (and standard errors) of each group of samples analyzed for each gene, same as in Table 2.

Reverse transcription polymerase chain reaction was performed to explore the expression levels of the selected *TALE* genes in samples from different tissues (sporophyte, spore filled sporangia, and gametophyte) belonging to the OJEN population of *V. speciosa*. We used the adenine phosphoribosyltransferase and squalene synthase genes as housekeeping genes (*HKG*s) (Table S1). The RQ values of the samples for each gene and the significance of differences (*p*-values) in *TALE* gene expression levels are indicated in Table 2 and Table 3, respectively. A graphic representation of the RQ values within each population/phase is in Figure 3.

While the *VsKNAT* genes 1 and 3 showed higher levels of gene expression in the sporophyte than in the gametophyte (significant only in the case of the *VsKNAT3* gene), the *VsKNAT4* and *VsKNAT6* genes showed significantly higher levels of gene expression in the gametophyte than in the sporophyte (Table 2 and Table 3, and Figure 3). On the other hand, the *VsBELL* genes were always more expressed in the sporophyte than in the gametophyte. The differences were significant for the *VsBELL4* and *VsBELL10* genes. The gene expression levels of the seven genes were always slightly lower in sporangia than in sporophyte, but only in one case, that of the *VsKNAT1* gene, was this difference significant. In order to discard the possibility of variation between populations, we analyzed the expression patterns of *TALE* genes also in the VALD population. We found equivalent differences for gene expression patterns between gametophyte and sporophyte in OJEN and VALD populations, and there were not significant differences for comparisons between OJEN and VALD sporophytes on one hand, and between OJEN and VALD gametophytes, on the other (not shown).

### 3.3. Expression Analysis of TALE Homeobox Genes in La Almoraima, a Population Composed Only by Independent Gametophytes

**Figure 4:**
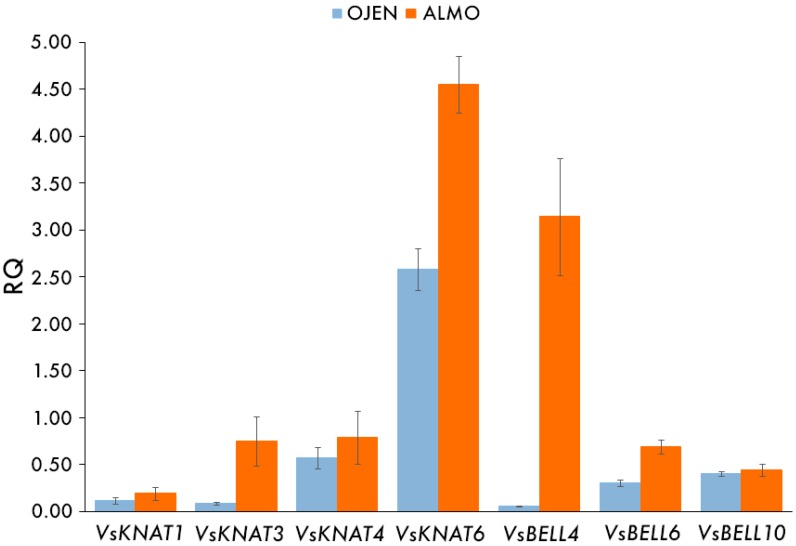
Graphic representation of the mean RQ values (and standard errors) of each group of samples analyzed for each gene, same as in Table 4. La Almoraima (ALMO), Canuto de Ojén Quesada (OJEN).

In order to investigate if the *TALE* gene expression patterns of the gametophyte of ALMO were differential from the populations with both sporophytes and gametophytes, we performed qRT-PCR in ALMO and OJEN gametophyte samples. The RQ values of the samples for each gene, and the significance of differences (*p*-values) in TALE gene expression levels are indicated in Table 4. A graphic representation of the RQ values within each population is in Figure 4. There is a significant increase of gene level expression of four genes in the ALMO population with respect to the OJEN population: *VsKNAT3*, *VsKNAT6*, *VsBELL4*, and *VsBELL6*.

## 4. Discussion

TALE homeodomain proteins are key transcription factors controlling the gametophytic and the sporophytic developmental programs [21,22,23,24,25]. Ferns, as the sister lineage of seed plants, are vascular plants of great importance for the study of plant evolutionary developmental biology [1,2,3,4,5,6,7]. In this context, *V. speciosa* is a species with specific features, which allows the existence of an interesting model of study of fern development. Specially, as we demonstrate in this paper, the existence of independent gametophyte populations allows us to analyze changes in gene expression levels of developmental genes involved in the transition between the two phases of this fern’s lifecycle. In this study, the examination of the two NGS assembled transcriptomes of *V. speciosa* revealed 22 contigs of *TALE* genes, 11 *KNOX*, and 11 *BELL* genes. The characterization and analysis of seven of these genes (four *KNOX* and three *BELL* genes) are of special interest, as it provides new information about the structure, the evolution, and the expression of *TALE* genes in ferns, a group of vascular plants for which genomic data are scarce [27,38,39,40]. BLAST search and sequence alignments of deduced amino acid sequences (Figure 1 and Figure 2) demonstrate that the seven selected genes encoded TALE proteins: 2 KNOX1 proteins and 2 KNOX2 proteins, as well as 3 BELL proteins. Sequence analyses demonstrated that these proteins are homologous to KNOX and BELL proteins of other ferns, as well as to this type of proteins in mosses, lycophytes, and seed plant.

Prior to the origin of land plants, a gene duplication in an ancestral *KNOX* gene generated the two classes of *KNOX* genes (*KNOX1* and *KNOX2*) [11,22,25,26,27]. The ancestral role of the *KNOX* and *BELL* genes was the regulation of sexual and zygote development as occurs in green algae and several fungi [24,25,32]. In the moss *P. patens*, both class I and class II *KNOX* genes are predominantly expressed in sporophytes, although *KNOX2* genes have also been detected in the haploid tissues, such as egg mother cells and mature eggs [24,58]. *KNOX1* genes are involved in cell proliferation during sporophyte development [25,32,58], while *KNOX2* genes are required to repress gametophyte developmental program in sporophytes, having a critical role in establishing an alternation of generations in land plants by the regulation of the gametophyte-to-sporophyte developmental transition [24,32,58]. Angiosperm class I gene function is slightly reminiscent to that of moss homologs, because both are involved in cell proliferation, whereas the neofunctionalization of class II genes was instrumental in the evolution of more complex multicellular diploid (sporophytic) generations in land plants, providing plasticity for the morphological evolution of land plant body plans [25,32]. In *Arabidopsis*, *KNOX1* activity promotes meristem maintenance and *KNOX2* activity promotes tissue differentiation [25]. In general terms, *KNOX1* genes in angiosperms are involved in the formation and maintenance of the shoot apical meristem (SAM), contributing to SAM function as well as to inflorescence and fruit development [59,60,61,62], and to flower patterning [18,22], whereas *KNOX2* genes are expressed in leaves, floral organs, seeds, and roots, having a broad tissue specificity compared to *KNOX1* genes. *KNOX2* genes confer opposing activities rather than redundant roles with *KNOX1* genes, and together, they act to direct the development of all above-ground organs of the *Arabidopsis* sporophyte [25]. The *KNOX* genes characterized to date in ferns are expressed in the sporophyte shoot apical meristems, but not in the gametophyte [27,38,39,40]. However, in *V. speciosa*, we find a differentiated role for each of the two genes belonging to each of the two KNOX classes. Thus, one *KNOX2* (VsKNAT3) is expressed predominantly in the sporophyte, with a residual expression level in the gametophyte, whereas one *KNOX1* (VsKNAT6) and one *KNOX2* (*VsKNAT4*) are expressed predominantly in the gametophyte, but they are also expressed in the sporophyte (at a lower level). Therefore, our analysis reveals a potentially important activity of both classes of *KNOX* genes in the maintenance and development of both the gametophyte and the sporophyte. The ALMO population is composed only of independent gametophytes that do not reproduce by sexual means, but they reproduce asexually. In this population, the expression levels of the *VsKNAT3* and *VsKNAT6* genes are increased, which supports an important role of these two genes in the control of the transition between the two phases of the lifecycle of this species.

In this context, the expression patterns in *V. speciosa* of the structurally and functionally related *BELL* genes appear relevant. These genes are expressed both in the sporophyte and in the gametophyte phases of this species. However, VsBELL4 and VsBELL10 are expressed at a higher level in the sporophyte than in the gametophyte, the expression of VsBELL4 being residual in the gametophyte, while the *VsBELL6* gene show similar expression levels in both phases. Interestingly again, as for the *VsKNAT* genes, things change in the gametophyte of the ALMO population, since the expression levels of VsBELL4 and VsBELL6 increase considerably. These data seem to also support also a relevant interactive role of *VsBELL* and *VsKNAT* genes in the alternation of generations in *V. speciosa*. The ancestral role in the life cycle progression of BELL proteins was inherited by land plants and is preserved in bryophytes [23,24,63]. In the moss *P. patens*, PpBELL1 activity is necessary for sporophyte development, it could be a master regulator for the gametophyte-to-sporophyte transition in *P. patens* and may trigger phase transition, embryogenesis, and asexual reproduction [23,63]. PpBELL1 and the rest of the *P. patens* BELL proteins can form heterodimers with each of the KNOX proteins of this species. Subsequently, KNOX–BELL complexes may induce the expression of additional *KNOX* genes. Similar TALE protein complexes function as transcriptional switches that regulate various developmental pathways in plants [18], such as SAM maintenance and inflorescence patterning in *Arabidopsis* [59,64], seed germination and early seedling development in *Arabidopsis* [65], or tuber formation and root growth in potato [17,20,66].

In conclusion, this study offers novel data and opens the door for future further research on the homeodomain proteins of fern VsKNAT3 (a KNOX2 class protein), as well as VsBELL4 and VsBELL10 proteins, among others, which may control the vegetative program. On the contrary, one KNOX1 protein (VsKNAT6) and one KNOX2 protein (VsKNAT4) seem important during the gametophyte phase development. Furthermore, they might be among the key regulators of the gametophyte-to-sporophyte developmental transition in populations with alternation of generations, since some of them (VsKNAT3, VsKNAT6, VsBELL4, and VsBELL6) are upregulated in the non-alternating population, and might be triggering the vegetative propagation of the gametophyte and the repression of the sexual development. In that case, we could propose that VsKNAT4 protein may be probably involved in sexual cell development while VsKNAT6 could favor the asexual reproduction of the gametophyte. We might then further propose that *VsKNAT1*, *VsKNAT3*, *VsKNAT6*, and *VsBELL* genes could be involved in the vegetative development of both the sporophytes and the peculiar gametophytes of *V. speciosa*.

In a larger context, this paper provides new information about the structure, the evolution, and the expression of *TALE* genes in ferns, a group of vascular plants for which genomic data are scarce. *TALE* genes belong to a very ancient homeobox gene superclass that encodes for key transcription factors controlling the gametophytic and the sporophytic developmental programs, as well as the gametophyte-to-sporophyte developmental transition that characterizes plants. There is little information on the role of *TALE* genes in ferns, and this study has found new insights on the potential role of both *KNOX* and *BELL* genes in the genetic control of the development of this fern species. Thus, several of these genes were found differentially expressed both in the sporophyte and in the gametophyte phases of *V. speciosa*. Furthermore, the existence of independent gametophyte populations of this species allowed us to analyze changes in gene expression levels of the developmental genes that might be involved in the transition between the two phases of this fern’s lifecycle.

## Figures and Tables

**Table 1:** Sequence identity of each *Vandenboschia speciosa KNAT* (VsKNAT) and *BELL* (VsBELL) proteins to different KNOX and BELL proteins from different plant species, according to the Basic Local Alignment Search Tool BLAST analysis.

	Ferns ^(1)^	Lycophytes ^(2)^	Moss ^(3)^	Spermatophytes
VsKNAT1	63–69%	54%	--	44–60%
VsKNAT3	49%	43–45%	39–40%	48–88%
VsKNAT4	43%	41–46%	39–45%	46–49%
VsKNAT6	70–75%	--	--	51–65%
VsBELL4	--	40–59%	46–48%	37–43%
VsBELL6	--	45–52%	45–46%	45–52%
VsBELL10	--	47%	48–50%	47–54%

^(1)^ *Ceratopteris richardii* and *Elaphoglossum peltatum*; ^(2)^ *Selaginella moellendorffi* and *Selaginella kraussiana*; ^(3)^ *Physcomitrella patens*.

**Table 2:** Relative quantification (RQ) values of the transcripts ± standard error (parenthesis) for each group of samples analyzed by qPCR. The value for each sample represents the mean RQ value of five individuals taken from the same population and collecting period.

Developmental Phase	*VsKNAT1*	*VsKNAT3*	*VsKNAT4*	*VsKNAT6*	*VsBELL4*	*VsBELL6*	*VsBELL10*
Sporophyte	0.43 (0.06)	1.98 (0.35)	0.23 (0.04)	0.24 (0.03)	0.85 (0.31)	0.40 (0.06)	1.11 (0.30)
Sporangia	0.23 (0.04)	1.68 (0.40)	0.19 (0.06)	0.26 (0.10)	0.46 (0.06)	0.40 (0.04)	0.59 (0.08)
Gametophyte	0.21 (0.07)	0.21 (0.07)	1.73 (0.36)	1.46 (0.08)	0.05 (0.01)	0.31 (0.03)	0.40 (0.03)

**Table 3:** Estimated *p*-values of the non-parametric Mann–Whitney *U* test for the significance of differences between the relative quantification (RQ) values of the transcripts in the groups of samples. Asterisk indicates significant differences between the expression levels as detected by qPCR.

Developmental Phase	*VsKNAT1*	*VsKNAT3*	*VsKNAT4*	*VsKNAT6*	*VsBELL4*	*VsBELL6*	*VsBELL10*
Sporophyte–Gametophyte	0.076	0.009 *	0.009 *	0.009 *	0.009 *	0.175	0.028 *
Sporangia–Gametophyte	0.465	0.009 *	0.009 *	0.009 *	0.009 *	0.117	0.047 *
Sporophyte–Sporangia	0.047 *	0.465	0.601	0.917	0.602	0.917	0.251

**Table 4:** The two first lines indicate the relative quantification (RQ) values of the transcripts ± standard error (parenthesis) for each group of samples analyzed by qPCR. The value for each sample represents the mean RQ value of five individuals taken from the same population (OJEN or ALMO) and collecting period. The last line represents the estimated *p*-values of the non-parametric Mann–Whitney *U* test for the significance of differences between the relative quantification (RQ) values of the transcripts between both samples. Asterisk indicates significant differences between the expression levels as detected by qPCR.

Population	Phase	*VsKNAT1*	*VsKNAT3*	*VsKNAT4*	*VsKNAT6*	*VsBELL4*	*VsBELL6*	*VsBELL10*
OJEN	Gametophyte	0.11 (0.04)	0.09 (0.01)	0.57 (0.11)	2.58 (0.22)	0.05 (0.01)	0.31 (0.03)	0.40 (0.03)
ALMO	Gametophyte	0.19 (0.07)	0.75 (0.26)	0.79 (0.28)	4.55 (0.30)	3.14 (0.63)	0.69 (0.07)	0.44 (0.07)
OJEN–ALMO	Gametophyte	0.347	0.009 *	0.917	0.009 *	0.014 *	0.014 *	0.806

## Supplementary Materials

The following are available online at www.mdpi.com/2073-4425/8/10/275/s1, Table S1: Sequence of the forward and reverse primers used in this work. Figure S1: Partial nucleotide sequences of the *VsKNOX* and the *VsBELL* genes studied in this paper.
