# Supplementary material for: Identification and Characterization of TALE Homeobox Genes in the Endangered Fern Vandenboschia speciosa
Source: Genes (Basel). 2017 Oct 17;8(10):275. doi: 10.3390/genes8100275 (PMC5664125; doi:10.3390/genes8100275)
Supplement: Supplementary file 1 [file genes-08-00275-s001.zip › Supplementary_material/Table S1.pdf]

| Gene | Forward | Reverse |
| --- | --- | --- |
| VsKNAT1 | 5’ TCAAGCGAAATCAGAACACG 3’ | 5’ TATGCTGTAATGAGTCGCGG 3’ |
| VsKNAT3 | 5’ CAAGCCATTACAGCCTCCAT 3’ | 5’ GATCGATGCACAAATCAACG 3’ |
| VsKNAT4 | 5’ TCTTCTTCCGTGGGATATGG 3’ | 5’ CGTGATGAGCTCAAAATGGA 3’ |
| VsKNAT6 | 5’ CAGCAGAGCCACGTCATTTA 3’ | 5’ GGCGGAGCTAAGGAAGAAGT 3’ |
| VsBELL4 | 5’CGTCGCTAACGAAAGAAATG 3’ | 5’AAGCTGAGCGTCACTCCATT 3’ |
| VsBELL6 | 5’ TTGCTAATGTTGCCTGTTGC 3’ | 5’ CCCTTATGAAGCCCGGTTAT 3’ |
| VsBELL10 | 5’ TTCCTTCATCCGTACCCAAA 3’ | 5’ TGTCACCAGTGGACCTTTGA 3’ |
| HKG-1 | 5’ GCTACCGTCGGAGAAGACAG 3’ | 5’ CCAAAGCAGCACACACAGTT 3’ |
| HKG-2 | 5’ TGTGGCAATCACCCTTCATA 3’ | 5’ CAAGGGTTCAAGAGCGAGAG 3’ |

**Table S1.** Sequence of the forward and reverse primers used in this work. HKG-1: Housekeeping Gene 1: Adenine phosphoribosyltransferase. HKG-2: Housekeeping Gene 2: Squalene synthase
