# Supplementary material for: Identification and Characterization of TALE Homeobox Genes in the Endangered Fern Vandenboschia speciosa
Source: Genes (Basel). 2017 Oct 17;8(10):275. doi: 10.3390/genes8100275 (PMC5664125; doi:10.3390/genes8100275)
Supplement: Supplementary file 1 [file genes-08-00275-s001.zip › Supplementary_material/Figure S1.pdf]

>VsKNAT1

GAGGTTTCGGACGCCGAATCTCTTAGGGCTAAGATAATGGCGCATCCAAGCTTCCCGCGACTCA
TTACAGCATACACGAATTGTCAAAAGGTTGGTGCACCGCCGGATGTTGTTACAAGGTTGGATAT
ACGTTTAGACGAGCTGAGTAAAGATGGTCAGAACTTCGAACCCATGAAATCATCCAGCGCCGTA
GGAGAAGACCGAGAGCTCGACGACTTCATGGACACGTACTGTGATGTGTTGCAAAAGTACCATG
AGGAGCTGACAAGGCCCTTTAAGGAAGCCATGGCCTTCTTCAGAAAAATCGAATTGCAACTGAA
CAGTCTTGGCAAAGGCAGCAGCTTAAGACTCTCTCAATCAGTAGATGAGAAAATGGATGCCAAT
GGATCGTCCGACGAGGAGGATGGGGGTTGTGGTGAGCTAGACTACCACGACCTGGATCCGCTGG
CGGAAGACCACAAAATCAAGGAGCTGCTTTTGCGAAAGTATAGCGGGTACATCAGCTCACTGAA
GCAAGAATTTTTGAAGAAGAAGAAAAAAGGAAAGCTACCGAAGGAAGCCAGGCAAAAACTTCTG
GACTGGTGGGGTGAGCACTACAAATGGCCGTATCCATCGGAGGCTGAAAAGGCAGCGCTGGCAG
AGTCAACGGGCCTTGATCAGAAGCAGATAAATAACTGGTTCATCAACCAAAGGAAGAGACATTG
GAAACCTTCTGAAGATATGCAGTACGTAGTAGTCGAAAGTCCCAGTAGT

>VsKNAT3

CAAGTTGCAAAGTTTAAAGCGCAGATTTTGTCACATCCTATGTACGAGCAGCTACTGGCAGAAC
ACGTTGGGTGCTTGCGTATAGCCACACCAGCGGATCATCTACAAACCATAGAAGCGCAACTTGC
GACGATGCAGCATGTAGCAGCTAGGTATGCCGGCATGCTTGGCAATGACACGCCTCTTTCTCAG
GACGAGAAGGAGGAGCTCGATCGCTTCATGGTAAGCTTCCTTCCAGTTGTTTTTCGCTTCCCTG
TAGGTCATTACGTTCAGCTCGCTGGAAACATCAC

>VsKNAT4

GAAACAGCGAGACTGAAATCGGAAATAATAAATCATCCTTTATACGAGCACGTGCTAGAAGCTC
ACGTATCGTGCTTGTGCGTCGGTTTACCAATGGATTGCATTCAAGCCGTGGAGTCTCAGCTTGC
TAACAGACACAGTGTCGCCATGAAGTATTGCATGCTGGGCAGAAACGAAGGTTTGGTGTTGGAC
AGAAGCAAAGAGATAGATGAGTTTATGAAAAATTATTTGCTCCTGTTGCACTCGTTTAAGAAGC
ATTTGGAAGAGCATTTTGTCCAACCTATGAAAGAGGCAATGGAAGCATATATGGAAATCGAACA
AACCTTTCAAGCATTTACAGGAGTGTCTTCCATAAATTTACCTACCACAAGCAAGGTGAACAAA
GAACTCGAAGATGATCTTCTTCATGGCAATGATGATTTCACTATGGACATAAAGATGCACTCTG
ACCAAGAGGATTCCCTATTGGAACAAGTTCGTGATGAGCTCAAAATGGAGTTGAAAGAGGAGTA
TTTCAAAAGCATAGCCAATATTAGGAAGGAAATCTTGCGGAAAAGACAAGTAGGAAAGCTTCAA
GGTGATACTGGTATTCTGAAACAATGGTGGAATGCTCATTTCGATTGGCCATATCCCACGGAAG
AAGATAAACAAAAAATGGTTGAAGCCACTGGTCTAGATCTCAAACAAATCAACAATTGGTTTAT
CAACCATCGTAAGAGAAATTGGAATGGGAGAGGTATTATTGATGGTGATACATTAAAAAGCTAT
CCAAAA

>VsKNAT6

GATTGCAAATGCACACTGCAAGAGACGTATTGTAATATTTTGCAGAAGTACTATGAGGAGCTTA
CAAAGCCATTTAAGGAAGCTGTGACCTTCTTCAGGAAAATAGAGCTACAGTTAAACACACTTAG
CAAAGGCAGCTTGACATTATCTCAAATAGAAAAAGAACTATCCCGAGTTTATCAAGTTACAAAA
AATAGCCG

>VsBEL4

CTAGACGCCAAATTCTCTCACTCAGGCACCTTAGGTTTACTAAGGAATTCCAAGTATCTTCGAG
CAGCACAGCAGGTTCTTGATGAATTCTGCCACGTTGGAAGGAGTGAGTTTATGAGCCTGCAGCC
TAATCAGGCGGACCAAATATCTCAAGCGTGGATGAAAAGGGGATTCACCCGAGTAGTTGAGGAT
AAAATAGACGATCAAGCTATTCATCCAGAGTCATTAGACCAAAACAGATCTCCGGTATCCATCT
CCGTGCGGCTTCCAATTCGACCAGAACCGAGCCCTATGCAATTGTCTGCAGATGAGAGGAGCCA
TCTGCAGTTGAAGAAAGGGCGACTCATTGGAATGGTTGAGGAGCTGGACGGAAGATATAGACAA
TACCGCGATCAAATGCAATTGGTAATCACTTCCTTTGAATCTGGTACTGGACTCGGTGCGGCTG
CGCCCTACACAACTTTGGCTCGGCAAGCTATGTCAAAGCGCTTTCGGAGCTTGAGAGACTCGGT

AAGTGATCAGATACAGAAGGTGTGCAAAACACTTGGGGAGGAGCTATCGAACGTGCCGATACTG
AACCGAGGAGAAACCCCTAGGCTGCGGGTTTTGGACCAGCGTTTACGGTACCAGAGAGCTTTGC
AGCAAATCGGTATGCTACAGCAGCAGGCGTGGAGGCCTCAGCGAGGATTGCCGGAGCGATCTGT
GTCTGTGCTCAGGGCTTGGCTGTTTGAGCATTTCTTGCACCCGTACCCCAAAGACTCAGAGAAA
CTTATGCTTGCTCGGCTAACGGGCCTCTCGAGAAGCCAGGTTTCCAACTGGTTCATAAACGCTC
GAGTTCGACTCTGGAAACCCATGGTAGAGGAGATGTACGTTGAGGAG

>VsBEL6

TCCATGCTCGACAATGGAGTTGCTCTTGGCTTAGATAGCGACAATTCACATGGTGCTGCCAGCT
CTCGTCAGGGCCTATCCCTCTCTCTTTCACCACAGCAGCATCCAGTTCAAATGCAGTACTATAG
CGTGCACCCCAATGATTCAAGCGTAAGCAGTTGTGCAGGATTAGCTCATGTCAACGCGGATCAG
AACAACGAAACTAGGGGGGGGAGATTTCCAAGTAAATGGATAGGAAATTTGAGCCAATTCAGAG
CATTATCTCGTGATGGCGTGCTGGCAACAGGTTTTCAGAATCCCATGAATGATACTAGCAGCAG
GCATAGGCAACCGGATGCAAACGTTCCAACGCTAGTAGGTTTTGGAGGTCCTCTCTCTTGTTCG
AAGTACTTGAAACCAGCACAGGAAGTTCTTGATGAAGTGGTGAGTGTTCAACGTGGAATCAAAG
GTAGTTCCTCAAGGCATTCGAAATTACCAGAGTCTTGGATGGCATCTAGCTCTTTTTTAGATGG
TGCTTTTGCAAGAGAGAGAAGTTTACCTGAGATTCTCAGTGTTGCGAAAGAAGGAATATCCTGT
ACAACGTCAATGCCAAGTGCTGCCTCTGGTCCCGTTCAATATTCTTCGAATGTGTCTGAGCTTT
CACAACAAGGAAGGCAAGAATTTCAAATGAAGAAAGCAAAGCTGTTGGCAATGCTGGACGAGGT
CGATCGGCGTTACAAGCAGTATTACGGTCAAATGCAAGTGGTTGTCGATTCTTTCGAATCTGCG
GCTGGACCTGGTGTTGCCAAAATGTATACATCCCTTGCTCTTCAAACTATATCGAGGCATTTTC
GCTCCTTGAGAGATGCAATCAGCAGTCAAATTCGTGTTGCAAGCGCAGCGCTCAATGAGGAAAA
TCCCTCGTCTCCAGCGCTAGGGCGTGGAGAGGCATCGAGATTACGCTACGTGGACCAGCATTTC
CGCCAGCAACGAGCTCTGCAGCAGCTTGGAATGATGCAACAACTAGCGTGGCGCCCACAGAGGG
GCCTCCCTGCATTTTCGGTCTGGGTCGTTGTTGTGTGTTTATTTTTGCATTTTCTTCTTGCGTA
CCCAAAAGATGCAGACAAGATTATGCTGGCCAGACAGACCGGACTCACTCGAAATCAGGTTTCA
AACTGGTTTATCAATGCACGTGTACGACTTTGGAAACCCATGGTGGAAGAGATGTACTTGGAGG
AG

>VsBEL10

CCAGGATTAGGTCATGTTGCTGTTGAAGACAGTGCTAATCGTGGAGAGAGAGCTCCAAGCAAGT
GGATTGGGAGCTTAAGCCATTTCAGAGCCTGTTCACGAGATGGCCTATTAGGATCAGGCTTTCA
GAATTCCACGAATGATATTAACAGCAGGCACATGCACATGGAATCAAATGTTTCAACAGTGGTA
GGACCTGGAGGTCTTCTCACTGGTTCAAAGTACTTAAAAGCAGCGCAGCAGCTTCTTGACGAAG
TTGTGAGTGTTGCACGAGTAGTTAAAAGCAGTTCCTCCAAACATTTGAAACTGCAGGCATGGAT
GGCACCCAATTCATTTATGGATGGCTCGTTTGCAAGAAACGGAAGCCTACCTGAAGCGGTCGCC
ACTGCGAAAGAGGGGACAGCTGGCACCACATCAATTCCGAGCTTTGCTTCTGGTCCTGAACAGA
ACTCTTCAACTGTTATTGAACTAACACGGGAGGAGCGACAGGAGCTTCAAACAAAGAAAGCAAA
GCTGATTGCTATGCTTGACGAGGTGGATCGAAGGTACAAGCAATACTATAGTCAGATGGAAATG
GTCGTGACTTCTTTCGAATCTGCAGCGGGACTTGGTGCTGCTAAGACGTACACCGCACTGGCTC
TTCAGACAATATCAAGACATTTTCGTTGTCTGAGGGACGCTATTATCGGTCAAATTCGTGTCGC
AAGCAGAGCGCTTGGTGAAGAAAATTTCTCATCTCCTTGTTTGGGAAGGGGAGAGACACCTAGG
CTACGTTATCTCGATCAGCAGTTGCGCCAGCAACGAGCACTCCAGCAGCTTGGAATGATGCAAC
AACACGCCTGGCGTCCTCAGAGGGGCCTCCCCGAGCGCTCTGTCTCAATTCTCAGAGCTTGGTT
ATTTGAGCATTTCCTTCATCCGTACCCAAAAGACTCAGACAAGCTTACGCTGGCCAGACAAACG
GGACTAACAAGAAACCAGGTTTCGAACTGGTTTATCAATGCGCGGGTACGGCTCTGGAAACCGA
TGGTTGAAGAAATGTACCTGGAAGAG
